# Disruption of tumour-host communication by downregulation of LFA-1 reduces COX-2 and e-NOS expression and inhibits brain metastasis growth

**DOI:** 10.18632/oncotarget.10737

**Published:** 2016-07-20

**Authors:** Manuel Sarmiento Soto, Emma R. O'Brien, Kleopatra Andreou, Simon F. Scrace, Rasheed Zakaria, Michael D. Jenkinson, Eric O'Neill, Nicola R. Sibson

**Affiliations:** ^1^ Cancer Research UK and Medical Research Council Oxford Institute for Radiation Oncology, Department of Oncology, University of Oxford, Oxford OX3 7LE, UK; ^2^ Institute of Integrative Biology, University of Liverpool, Liverpool, L69 3BX, UK; ^3^ Department of Neurosurgery, The Walton Centre NHS Foundation Trust, Liverpool, L97LJ, UK

**Keywords:** brain metastasis, cellular adhesion molecules, LFA-1, COX-2, eNOS

## Abstract

Over 20% of cancer patients will suffer metastatic spread to the brain, and prognosis remains poor. Communication between tumour cells and host tissue is essential during metastasis, yet little is known of the processes underlying such interactions in the brain.

Here we test the hypothesis that cross-talk between tumour cells and host brain cells, through tumour cell leukocyte function associated protein-1 (LFA-1), is critical in metastasis development. Temporal expression of LFA-1 and its major ligand intercellular adhesion molecule-1 (ICAM-1) was determined in two different mouse models of brain metastasis. Marked upregulation of both proteins was found, co-localising with astrocytes, microglia and tumour cells themselves. Silencing of LFA-1 expression in MDA231Br-GFP cells prior to intracerebral injection resulted in > 70% reduction in tumour burden compared to control MDA231Br-GFP cells (*p* < 0.005, *n* = 5). Subsequent qRT-PCR analysis of brain tissue revealed significant reductions in COX-2, VEGF and eNOS from host brain tissue, but not tumour cells, in mice injected with LFA-1 knockdown cells (*p* < 0.0001, *n* = 5). Finally, expression of both LFA-1 and ICAM-1 was demonstrated in human brain metastasis samples.

The results of this study suggest LFA-1 as a new target in brain metastasis therapy and highlight the potential synergy with current anti-COX-2 and anti-NOS therapies.

## INTRODUCTION

Despite advances in the treatment of primary tumours, the incidence of secondary tumours (metastases) is increasing, particularly in the brain [1]. Brain metastases occur in over 20% of cancer patients, with the most common primary cancers to spread to the brain being lung, breast and melanoma.

A significant hurdle, however, to the development of effective brain metastasis therapies is the lack of *in vitro* and *in vivo* studies that accurately recapitulate the multistep pathogenesis. We have previously reported the development of *in vivo* models of breast cancer brain metastasis that enable investigation of the mechanisms involved in both the initial stages of seeding to the brain and downstream proliferation stages within the brain parenchyma [2, 3]. In those studies we have shown marked and early upregulation of specific subsets of cell adhesion molecules (CAMs) [3]. However, the contribution of CAM-mediated pathways to tumour growth within the brain parenchyma, following extravasation from the bloodstream, remains unclear. One molecule, in particular, that showed marked and consistent upregulation in our experimental models was intercellular cell adhesion molecule-1 (ICAM-1) [3]. This cell surface glycoprotein is typically expressed by both endothelial and immune system cells and its role in inflammatory processes has been widely described [4]. At the same time, leukocyte function associated protein 1 (LFA-1, also known as CD11a-CD18 and α_L_β_2_), a cognate ligand to ICAM-1 [5], was also strongly upregulated, particularly on tumour cells [3].

Involvement of LFA-1/ICAM-1 in the immune response against cancer cells has been indicated both *in vitro* [6] and, more recently, *in vivo* [7, 8]. In particular, LFA-1 has been extensively described as having an essential role in leukocyte extravasation at cancer sites [9], whilst other studies have suggested a requirement for LFA-1/ICAM-1 interactions between melanoma and endothelial cells to aid transmigration of tumour cells [10]. However, no studies to date have specifically considered the role of LFA-1-mediated pathways during metastasis growth within the brain, once extravasation across the blood-brain barrier (BBB) has occurred, and it remains unclear whether these are pro- or anti-tumorigenic.

In recent years, immunotherapy has gained credence as a strategy for cancer treatment, with multivariate approaches using adjuvant radio- and chemotherapy [11, 12]. Although not currently undergoing trials in cancer therapy, LFA-1 has been the object of intense study in a number of clinical trials [12], with different modes of application including antibody therapy (Odulimomab), small molecules (SAR1118) and siRNA approaches [13]. Taken together, these studies suggest that this particular integrin may be an attractive target in the clinic and may have, hitherto unexplored, potential in brain metastasis.

Based on the above findings, we hypothesised that signalling between LFA-1 on tumour cells and ICAM-1, or its alternative ligands ICAM-2, ICAM-3, ICAM-4 and junctional adhesion molecule-1 (JAM-1) [14, 15], contribute to successful tumour growth within the brain parenchyma, and that molecules within the LFA-1 signalling pathways may provide potential therapeutic targets in brain metastasis.

## RESULTS

### Expression of LFA-1 and ICAM-1 in mouse brain metastasis models

**Figure 1:**
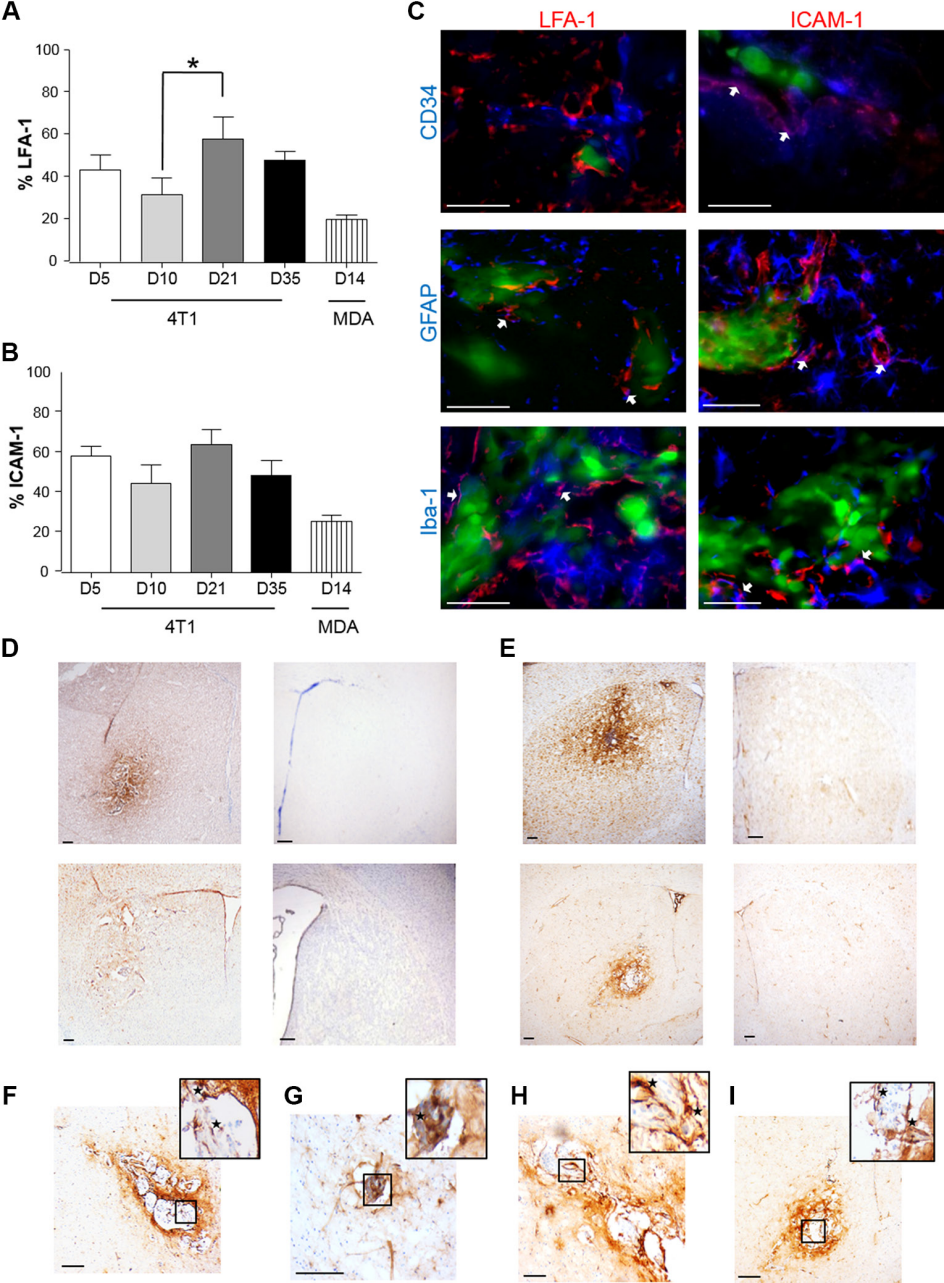
Time-course study of the percentage of expression of LFA-1 (A) and ICAM-1 (B) within the tumour area, in BALB/c and SCID mice injected intracerebrally with 5 × 10^3^ 4T1-GFP or MDA231Br-GFP cells, respectively Data were acquired at days 5, 10, 21 and 35 for the 4T1-GFP model, and at day 14 for the MDA231Br-GFP model (*n* = 6 per time point). Results are expressed as the percentage area of LFA-1 expression (A) and ICAM-1 (B) as a function of the total tumour area for each time-point. A minimum of 8 sections per animal (*n* = 6), evenly distributed through the striatum, were used to quantify the presence of each CAM. Statistical significances across the 4T1-GFP time-course study were determined by one-way ANOVA, with Tukey post-hoc tests. (**C**) Triple-colour fluorescence images showing co-localisation between LFA-1 and ICAM-1 (red) in the syngeneic (4T1-GFP) mouse model and different cellular markers (blue): CD34 for endothelial cells, GFAP for astrocytes and Iba-1 for microglia. Tumour cells were GFP positive (green). Magenta (white arrows) indicates co-localisation between CAMs and cellular markers (arrows). Scale bars = 50 μm. (**D**–**E**) Representative photomicrographs showing immunohistochemical detection of LFA-1 (D) and ICAM-1 (E) at day 10 in BALB/c mice injected with 4T1-GFP cells (upper row) and at day 14 in SCID mice injected with MDA231BR-GFP cells (lower row); injected (left) and control (right) hemispheres shown. Scale bars = 200 μm. (**F**–**G**) Representative photomicrographs showing in higher resolution the expression of LFA-1 in BALB/c injected with 4T1-GFP cells (F) and SCID mice injected with MDA231BR-GFP cells (G). Scale bars = 100 μm. (**H**–**I**) Representative photomicrographs showing in higher resolution the expression of ICAM-1 in BALB/c injected with 4T1-GFP cells (H) and SCID mice injected with MDA231BR-GFP cells (I). Scale bars = 100 μm.

In the syngeneic 4T1-GFP model, increased expression of both LFA-1 and ICAM-1 was observed throughout the time-course and exhibited similar stable patterns of expression (Figure 1A and 1B). Both CAMs co-localised with astrocytes and microglia, whilst only ICAM-1 co-localised with endothelial cells. (Figure 1C and Table 1). Similar expression of LFA-1 and ICAM-1 was observed in the MDA231BR-GFP model at 14 days after tumour induction to that seen at 10 days after tumour induction in the 4T1-GFP model (Figure 1D–1I); expression of both CAMs was evident not only within the tumour area, but also spreading beyond the tumour margins throughout the striatum (Figure 1D–1E). No detectable expression of either ICAM-1 or LFA-1 was seen in the contralateral hemisphere (Figure 1D–1E) or in vehicle-injected mice (Supplementary Figure S1).

**Table 1:** Summary of biomarker expression and co-localisation with endothelial cells, astrocytes, microglia and tumour cells in the syngeneic 4T1-GFP model

	% Expression	Endothelium	Astrocytes	Microgila	TC
**LFA-1**	33.2 ± 10.4	–	+	+	+
**ICAM-1**	44.5 ± 7.7	+	+	+	+

The first column shows the area of expression (mean ± SEM) for each adhesion molecule within the tumour area, observed at day 21 post-injection. A minimum of 8 sections per animal (*n* = 6 per time-point), evenly distributed through the striatum, were used to quantify the presence of each CAM within the tumour area; expression was quantified as the percentage of tumour area covered using an unbiased algorithm in imagescope, which detects positive brown pixels. The remaining columns indicate co-localisation of each adhesion molecule with endothelium (CD34), astrocytes (GFAP), microglia (Iba-1) and tumour cells (TC; GFP).

### LFA-1/ICAM-1 expression in human brain metastasis samples

**Figure 2:**
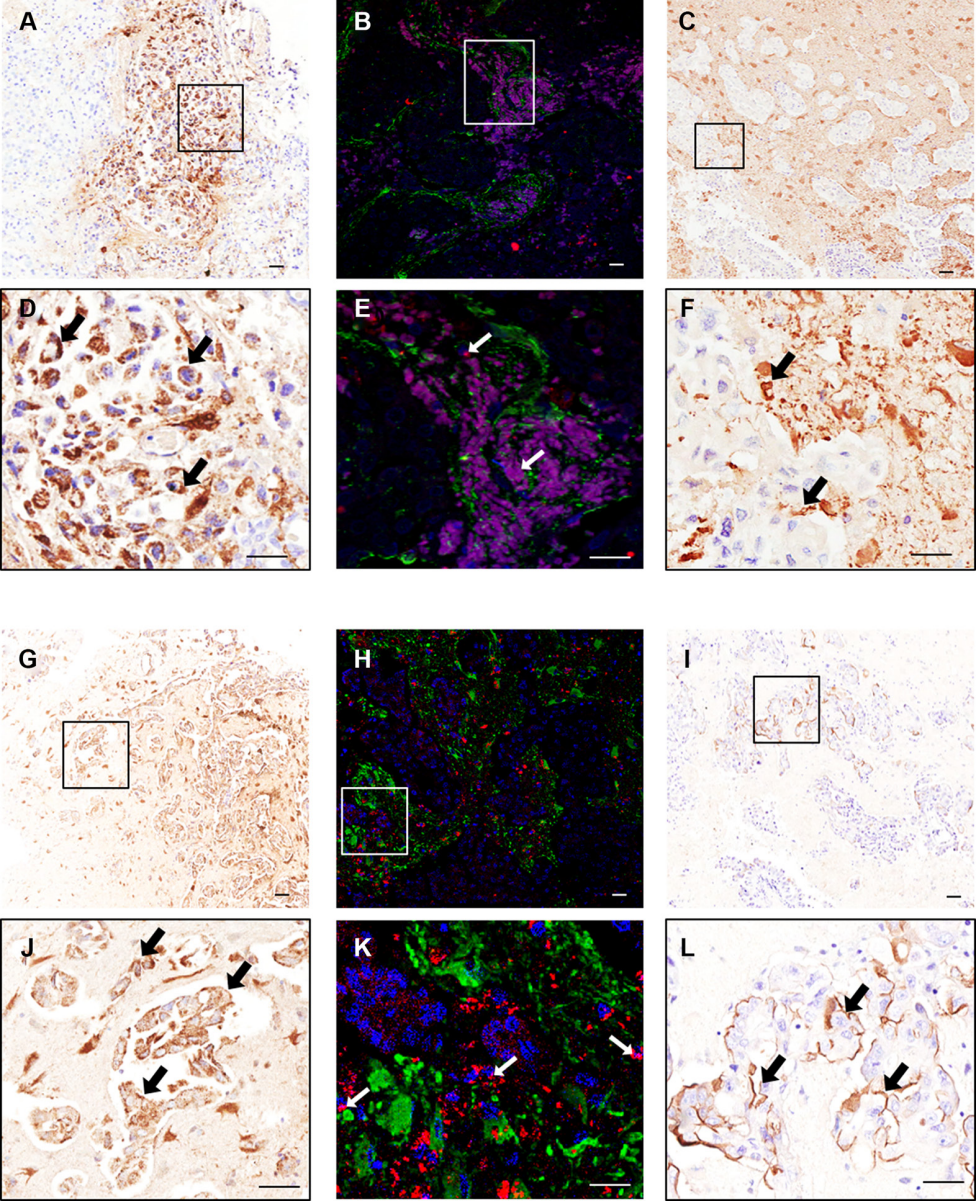
Photomicrographs of human breast carcinoma (**A**–**F**) and lung adenocarcinoma (**G**–**L**) brain metastasis resections stained immunohistochemically against LFA-1 (A–B and G–H) and ICAM-1 (C and I); higher magnification images from boxes shown in D–F and J–L. Scale bars = 50 μm. Widespread expression of LFA-1 is evident on tumour cells (brown staining; arrows, D and J) whilst ICAM-1 is evident on the intersection between tumour cells and brain parenchyma (brown staining; arrows, F and L). Immunofluorescence images of human breast cancer brain metastasis (B, E) and lung adenocarcinoma brain metastasis (H, K) demonstrate co-localisation (arrows) of LFA-1 (red) with tumour cells (blue; DAPI nuclear stain). Surrounding astrocytes stained green. Scale bar = 50 μm.

To determine the clinical relevance of the above findings, brain metastasis samples from breast carcinoma patients were assessed for hLFA-1 and hICAM-1 immunostaining. Additionally, brain metastasis samples from lung adenocarcinoma patients were also immunostained to determine the broader applicability of LFA-1 as a target in brain metastasis. In breast cancer patients, marked LFA-1 expression was evident within and around brain metastasis foci (Figure 2A–2B) whilst ICAM-1 was found predominantly around the micro-metastasis with a more widespread pattern (Figure 2C); as for the mouse models, expression of LFA-1 appeared to primarily co-localise with tumour cells (Figure 2D–2E), and ICAM-1 seemed to be expressed in more brain cell types (Figure 2F). At the same time, in lung adenocarcinoma samples, LFA-1 expression was also clearly upregulated in metastatic regions (Figure 2G–2H and 2J–2K), whereas ICAM-1 was detected primarily at the intersection between tumour cells and brain parenchyma (Figure 2I and 2L). All samples showed positive staining for both LFA-1 and ICAM-1.

### Effect of LFA-1 knockdown *in vitro* and *in vivo*

**Figure 3:**
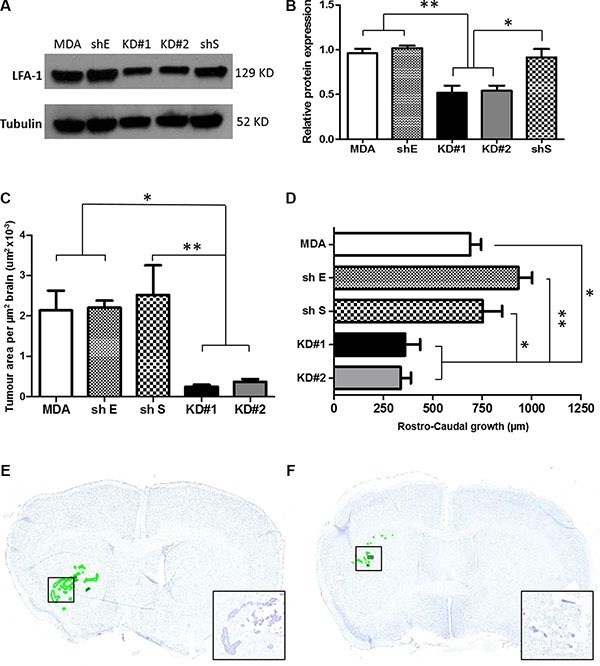
(**A**) Western blot showing the levels of LFA-1 protein expression in the five different experimental groups. Parental MDA231Br-GFP (MDA), scrambled shRNA (shS), empty shRNA (shE) and two different clones with significant reduction of LFA-1 expression, KD#1 and KD#2. (**B**) Quantitation of the mean intensity of each band with respect to the parental MDA231Br-GFP. Band densitometry was normalised to the intensity of tubulin bands and quantified across three independent experiments. Statistical significance was determined by one-way ANOVA, with Tukey post-hoc tests. *p < 0.05 and **p < 0.01. (**C**) Quantitation of tumour growth in animals injected intrastriatally with parental MDA231Br-GFP cells (MDA), control knockdown cells (empty cassette, shE; and scramble cassette, shS) or LFA-1 knockdown cells (KD#1 and KD#2) (*n* = 5 per group). Growth is expressed as tumour area/μm^2^ brain area analysed. Statistical significance determined by one-way ANOVA, with Tukey post-hoc tests. *p < 0.05 and **p < 0.01. (**D**) Quantitation of rostro-caudal tumour growth through the striatum in the same animals as for (C). Statistical significance determined by one-way ANOVA, with Tukey post-hoc tests. *p < 0.05, **p < 0.01. (**E**–**F**) Representative photomicrographs of brain sections from animals injected intracerebrally with parental MDA231Br-GFP (MDA, E) or KD#1 (F) cells; sections stained with cresyl violet and tumour foci are circumscribed in green. Inset shows higher magnification of the tumour colonies in boxed regions.

To determine the role of LFA-1 on tumour cells during metastatic growth in the brain, LFA-1 expression was knocked down in the MDA231Br-GFP cells by shRNA transfection. Given the concordance of CAM expression in the two experimental models, the human cell line was used for subsequent studies to enhance clinical relevance. Quantitatively, similar LFA-1 expression levels were evident *in vitro* in the parent MDA231Br-GFP (MDA) cells and those transfected with control shRNA plasmids (shS and shE), whilst cells transfected with the two anti-LFA-1 shRNA plasmids showed a significant drop in LFA-1 expression (~50%, *p* < 0.05–0.01, KD#1 and KD#2; Figure 3A–3B). No changes in either *in vitro* growth rates or cell activity were detected in any experimental cell line with respect to the parental MDA231Br-GFP cells (Supplementary Figure S2).

In the *in vivo* studies, mice injected with MDA, shS or shE cells exhibited similar tumour growth patterns (Figure 3C and 3D), whilst the animals injected with the LFA-1 knockdown cells (KD#1 and KD#2) showed significantly reduced tumour growth (Figure 3C–3F). Decreased growth was evident both in terms of tumour area (~70%, *p* < 0.05; Figure 3C) and rostro-caudal growth (< 50%, *p* < 0.05–0.01; Figure 3D). In order to elucidate whether the LFA-1 knockdown resulted in prolonged tumour growth inhibition, rather than just an initial suppression, a later time point (day 21 post-tumour cell injection) was assessed. Notably, a similar pattern of reduced tumour area was evident in the animals injected with LFA-1 knockdown cells (KD#1 and KD#2) in comparison to parental MDA231Br-GFP (MDA) and control knockdown animals (shE and shS; Supplementary Figure S3).

### Effect of LFA-1 knockdown on LFA-1 signalling pathways *in vivo*

**Figure 4:**
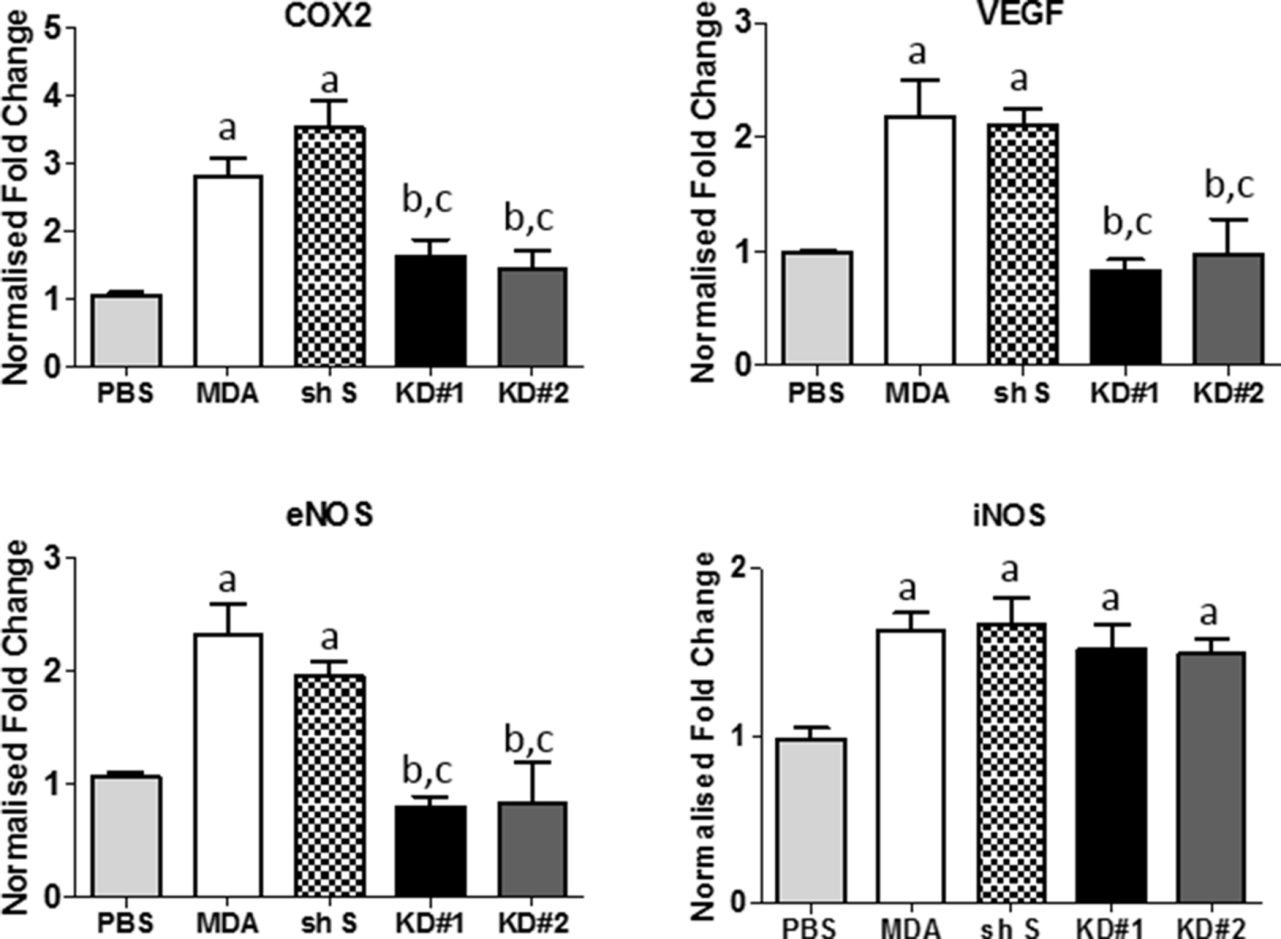
Quantitative reverse transcription PCR analysis of mouse gene expression in the striatum of mice injected with either PBS or one of the MDA231Br-GFP clones (*n* = 5 per group) Statistical significance determined by one-way ANOVA, with Tukey post-hoc tests. Multiple Comparison Test: a = significant compared to the PBS group; b = significant compared to the control MDA231Br-GFP group; and c = significant compared to the shS group. *p < 0.05 – 0.001.

The intercommunication between LFA-1 on tumour cells and ICAM-1 (or alternative ligands, ICAM-2/ICAM-3/ICAM-4/JAM-1) on other cells in the local vicinity triggers signalling through several pathways on each side [16]. Therefore, we investigated the expression of a panel of different genes that have previously been implicated either in LFA-1/ICAM-1 signalling in other tumour types or in metastatic spread of breast cancer [17–20]. These genes were assessed for both host tissue (mouse) and tumour cell (human) origin. Quantitation of mRNA levels in the injected striatum of each experimental group indicated that mouse COX-2, VEGF and endothelial NOS (eNOS) were all significantly reduced in animals injected with KD#1 or KD#2 compared to both the MDA and shS groups (Figure 4A–4C), and were not significantly different to the PBS injected controls. Interestingly, inducible NOS (iNOS) levels were similar for all groups injected with MDA231Br-GFP cells (MDA, shS, KD#1 and KD#2), irrespective of LFA-1 knock down, and in all cases were significantly elevated from control levels (Figure 4D). Mouse TGF-β showed no changes in any groups injected with tumour cells compared to PBS-injected controls (data not shown).

In contrast to the changes in mouse genes observed, no changes were evident in any of the human genes studied between mice injected with parental MDA231BR-GFP cells and knock-down clones; TGF-β, VEGF, eNOS, iNOS and COX-2 (Supplementary Figure S4A–S4E). Whilst we cannot exclude the possibility of changes below the sensitivity threshold of the experiment, we are confident that no substantial changes in human gene expression occurred since we were able to detect changes in human vimentin (Supplementary Figure S4F) in the MDA and shS groups, compared to the PBS injected controls. Assessment of human CXCL12 and mouse CXCR4 mRNA further confirmed that the hCXCL12/mCXCR4 axis was not altered by disruption of LFA-1 interactions with the brain environment; no changes evident in any of the groups (Supplementary Figure S4G–S4H).

### Cellular localisation of COX-2

**Figure 5:**
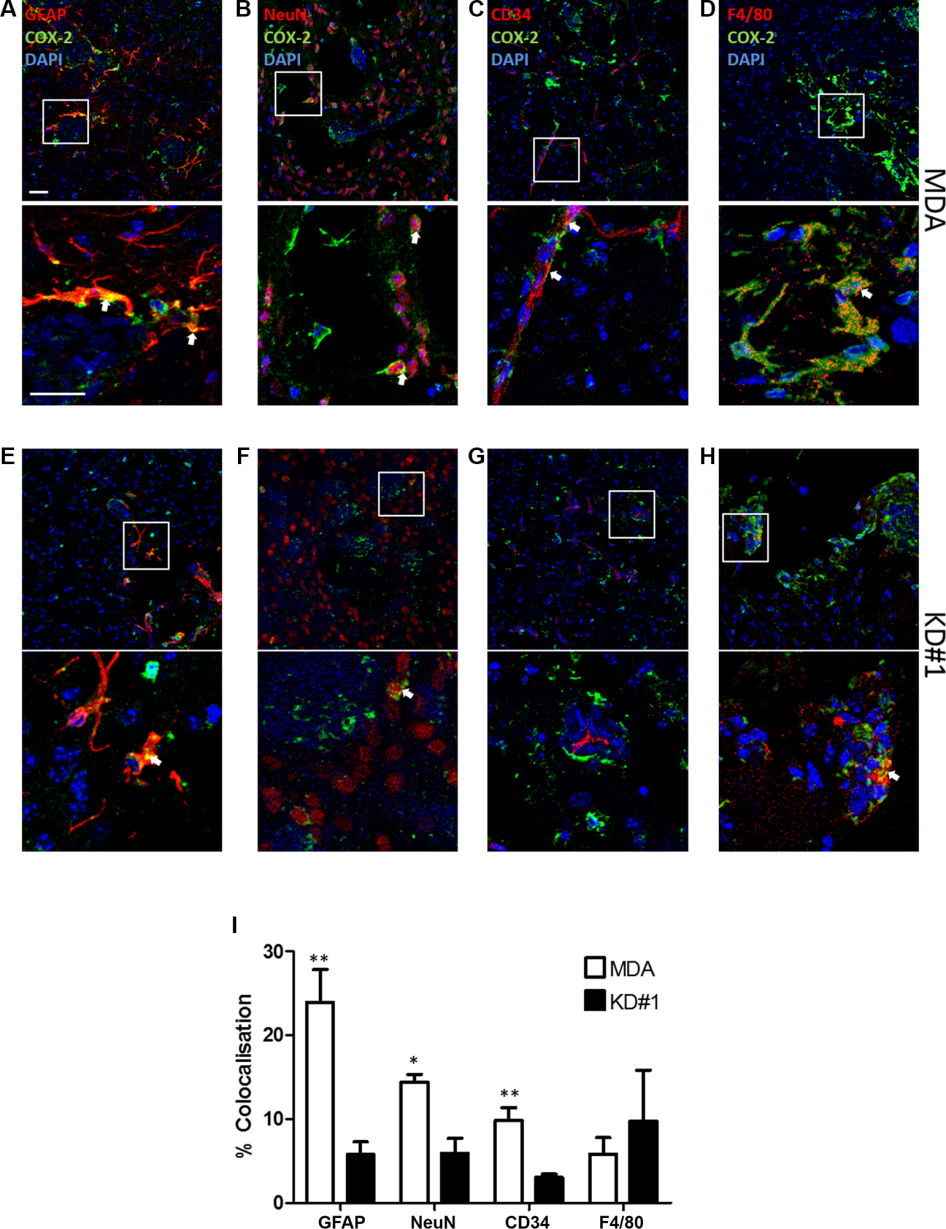
Immunofluorescent images of brain sections from animals injected with either MDA231Br-GFP (MDA) or LFA-1 knockdown (KD#1) cells showing COX-2 (green) co-localisation (arrows) with different brain cell populations in red: (A, E) astrocytes, GFAP; (B, F) neurons, NeuN; (C, G) vessels, CD34; and (D, H) macrophages/microglia, F4/80 Cell nuclei are stained with DAPI (blue). Scale bars = 100 μm (**I**) Quantitative analysis of COX-2 co-localisation with different brain cell populations in animals injected with either MDA231Br-GFP (MDA; white bars) or LFA-1 knockdown (KD#1; black bars) cells (*n* = 4). Statistical significance determined by one-way ANOVA, with Tukey post-hoc tests. **p < 0.01, ***p < 0.005.

To confirm the reduction in expression of COX-2 within the tumour environment following LFA-1 knockdown in tumour cells, and to determine its cellular origin, serial co-localisation of COX-2 with astroglia, microglia, endothelial cells and neurons was assessed. In mice injected with the parent MDA231Br-GFP cells, COX-2 predominantly co-localised with astrocytes (Figure 5A, 5I), with lower levels of co-localisation evident on neurons (Figure 5B, 5I), endothelial cells (Figure 5C, 5I) and microglia (Figure 5D, 5I). In animals injected with LFA-1 knockdown tumour cells, the levels of COX-2 were significantly reduced in astrocytes (~80% reduction; Figure 5E, 5I), neurons (~65% reduction; Figure 5F, 5I) and endothelial cells (~60% reduction; Figure 5G, 5I) compared to animals injected with the parent MDA231Br-GFP cells. In contrast, levels of COX-2 in microglia remained unchanged in all tumour-cell injected animals (Figure 5H, 5I). For the most part, COX-2 expression was evident in cells in direct contact with tumour cells. In some cases, expression appeared to extend to cells in close proximity to the tumour margin, but this may be an artefact arising from analysis of single sections in which the location of tumour cells in adjacent sections is unknown.

### *In vivo* assessment of NO release and p53 quantitation

**Figure 6:**
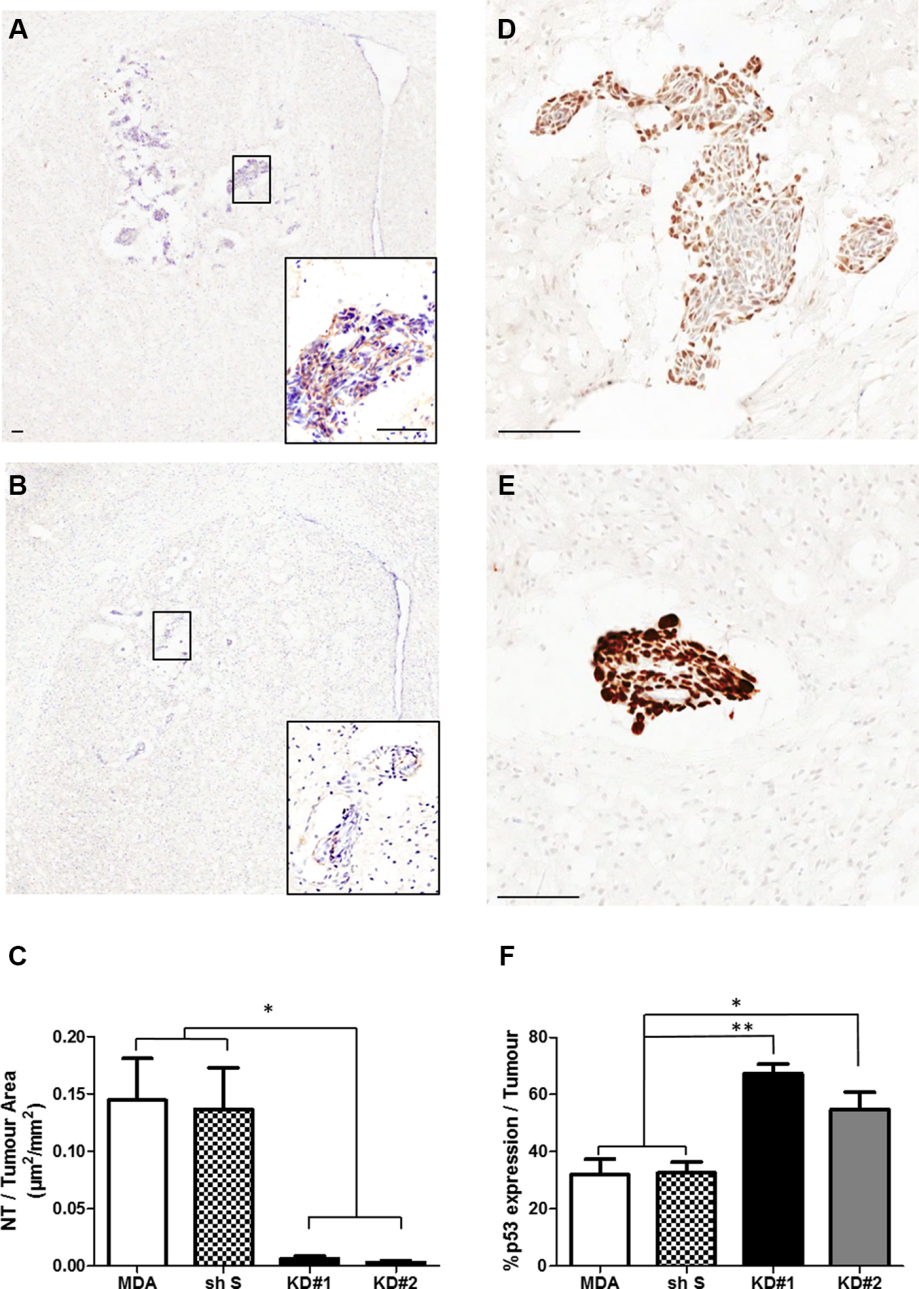
(**A**–**B**) Photomicrographs of brain sections showing NT staining within the striatum from a representative animal in each group: MDA (A) and KD#1 (B). Scale bars = 100 μm. (**C**) Quantitative analysis of the area of nitrotyrosine (NT) staining per mm^2^ tumour area in animals injected with MDA231BR-GFP cells (MDA), control knockdown cells (sh S), or LFA-1 knockdown cells (KD#1 and KD#2) at day 14 after intracerebral injection (*n* = 4). Statistical significance determined by one-way ANOVA, with Tukey post-hoc tests; *p < 0.05. (**D**–**E**) Photomicrographs of brain sections showing p53 staining within the striatum from a representative animal in each group: MDA (D) and KD#1 (E). Scale bars = 100 μm. (**F**) Quantitative analysis of p53 expression within the tumour area in animals injected with MDA231Br-GFP cells (MDA), control knockdown cells (shS), or LFA-1 knockdown cells (KD#1 and KD#2) at day 14 after intracerebral injection (*n* = 4). Statistical significance determined by one-way ANOVA, with Tukey post-hoc tests: *p < 0.05 and **p < 0.01.

Changes in NO release during tumour growth were further assessed via NT analysis in four groups of animals: MDA, shS, KD#1 and KD#2 (Figure 6A–6B). MDA and shS groups showed similar levels of NT within the tumour area, whilst animals injected with KD#1 and KD#2 showed a significant reduction in NT expression (> 75%; Figure 6C).

Given the reported links between NO and p53 [21–23], the effect of reduced NO production on p53 expression following LFA-1 knockdown was also assessed (Figure 6D–6F). In contrast to NT levels, p53-DINP expression within the tumour area was significantly increased in mice injected with LFA-1 knockdown tumour cells (55-65% tumour area) compared to the MDA and shS groups (~30% tumour area; Figure 6F). In order to assess potential changes in p53 expression over time, a second group of animals was assessed at a later time point (day 21, *n* = 3 per group). Again, significantly greater p53 staining was evident in KD#1 and KD#2 groups (≥70% tumour area) than the MDA and shS groups (~40% tumour area; Supplementary Figure S5).

### *In vitro* assessment of NO and p53 expression

**Figure 7:**
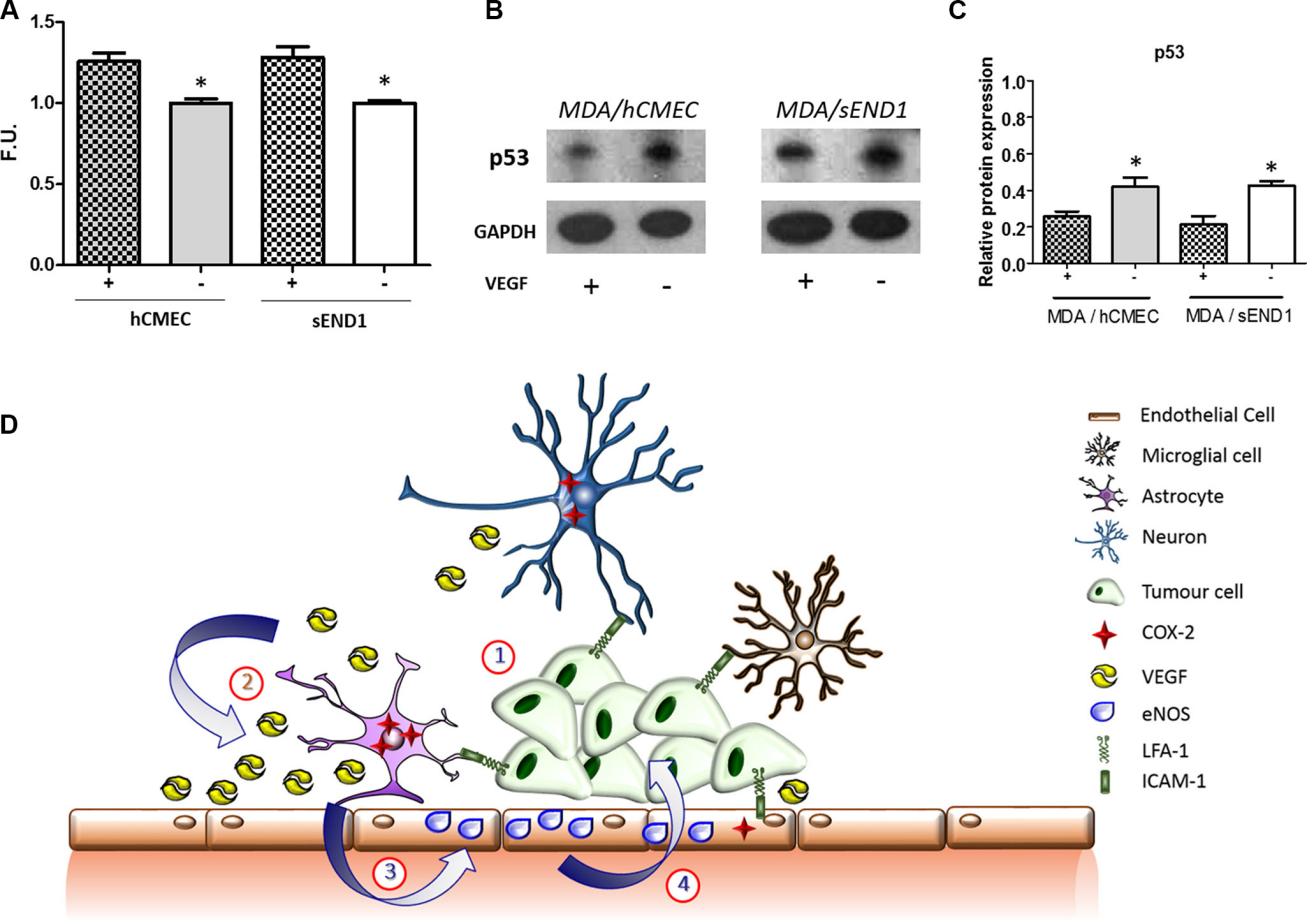
(**A**) Fluorescence quantitation of NO presence in human (hCMEC) and mouse (sEND1) endothelial cell lines +/− 48 h incubation with VEGF. *p < 0.05. (**B**) Western-blot showing p53 levels in MDA231Br-GFP cells after 72 h co-culture with hCMEC or sEND1 endothelial cells, respectively, +/− pre-activation of endothelial cells with VEGF. (**C**) Quantitation of p53 DINP1 levels in MDA231Br-GFP cells. Experiments were run in triplicate and statistical significance determined by t student: *p < 0.05. (**D**) Schematic illustrating putative activation of LFA-1 signalling pathways during metastatic growth in the brain. (1) The integrin LFA-1 on tumour cells interacts with its cognate ligand ICAM-1 (or alternative ligands ICAM-2, ICAM-3, ICAM-4 and JAM-1) on astrocytes, microglia, neurons and endothelial cells. (2) This interaction supports tumour growth by up-regulating COX-2 expression (predominantly in astrocytes) and consequent release of VEGF. (3) VEGF is taken up by endothelial cells and upregulates expression of endothelial NOS (eNOS). (4) The resultant increase in nitric oxide release impairs p53, potentially leading to enhanced tumour growth.

A significant increase in NO production by both mouse and human endothelial cells was evident following incubation with VEGF compared to control untreated cells (Figure 7A). Co-culture of MDA231Br-GFP cells with VEGF-treated human or mouse endothelial cells showed reduced levels of p53-DINP in the tumour cells compared to incubation with untreated endothelial cells (Figure 7B).

## DISCUSSION

Communication between tumour cells and the host tissue is essential to overcome the challenges of growing within the specialised environment of the brain. This work has demonstrated that inhibition of tumour-brain cell communication through downregulation of LFA-1 in tumour cells causes a significant reduction in metastasis growth. This inhibition of tumour growth was associated with reduced endothelial NO and increased tumour p53 production, which may result directly or indirectly from the reduction in COX-2 and VEGF expression observed in LFA-1 knockdown groups. Notably, resected human samples from both breast and lung cancer brain metastasis, the two most common primary ‘brain-seeking’ cancers [24], indicated the presence of LFA-1 and ICAM-1 on tumour cells and brain parenchyma, respectively, supporting the potential translation of these findings into brain metastasis therapies.

In this study, our aim was to specifically investigate processes involved in the post-extravasation stages of metastasis development in the brain and the role of tumour-host cell communication during this phase. To achieve this, we utilised a previously validated model of direct tumour cell injection into the brain parenchyma [25]. We achieved a single focal area of metastatic growth, which exhibited similar growth patterns to those observed following haematogenous dissemination to the brain (Supplementary Figure S6), and corresponding patterns of CAM expression [3].

Cell adhesion molecules have been shown to be essential in the bi-directional communication between metastatic cells and brain tissue [26–28]. However, the majority of previous studies on LFA-1 have focussed on its involvement in the immune system response to disease and injury. Our results indicate a novel role for LFA-1 as a key mediator between tumour and host cells during metastatic growth in the brain. Both ICAM-1 and LFA-1 have been shown to be involved in transendothelial tumour growth in melanoma *in vitro* models [10]. However, little is known of their role in the downstream proliferative steps of brain colonisation by metastatic tumour cells. In this study, LFA-1 expression was found in tumour cells, microglia and astrocytes, but not endothelial cells. In turn, as shown previously [3], ICAM-1 expression was evident on all cell populations. Given previous work indicating involvement of tumour cell LFA-1 expression in lymphoma [29] and liver metastasis [7], we hypothesised that owing to the presence of LFA-1 on breast cancer cells, knocking down LFA-1 expression in the tumour cells would impair interactions with host tissue cells, primarily through ICAM-1 interaction, and alter tumour colonisation in the brain. In accord with this hypothesis, animals injected intracerebrally with LFA-1 knockdown MDA231Br-GFP cells showed a significant reduction in tumour growth in comparison with the parent MDA231Br-GFP cells. These findings indicate a central role for LFA-1 signalling in brain metastasis and suggest new potential therapeutic targets.

To understand the downstream consequences of inhibiting binding between LFA-1 on tumour cells and its ligands, both on host brain cells and other tumour cells, we assessed expression of seven predominant genes in LFA-1-induced signalling pathways. Interestingly, no change in any of the human genes (tumour cell origin) studied was found, whilst expression of several mouse genes (host tissue origin) was evident. These findings suggest that LFA-1 knockdown in tumour cells had a greater effect on tumour-brain cell communication, than on tumour-tumour cell communication, although we cannot exclude the possibility that other genes in these pathways (including those in tumour cells) are involved. One important pathway that has been shown to be involved in breast cancer dissemination to distant organs is the CXCL12/CXCR4 axis [18, 20]; however, in our brain metastasis models neither of these genes showed significant changes. A number of reports have also suggested a role for both LFA-1 and ICAM-1 in VEGF production [17, 30, 31]. *In vitro* studies have shown that LFA-1 expression on tumour cells facilitates interactions between tumour cells and the environment, and contributes to increased release of VEGF through a COX-2 dependent mechanism [17, 32, 33]. In accord with those findings, the current study has demonstrated a marked reduction in COX-2 expression, primarily in astrocytes, together with reduced VEGF gene expression in animals in which LFA-1 signalling was disrupted. At the same time, reduced VEGF production was associated with downregulation of eNOS and NO release, supporting the concept of VEGF-driven eNOS activity, as previously reported [33]. In comparison, inducible NOS (iNOS) was significantly elevated in all groups compared to vehicle injected controls, and no difference was observed following LFA-1 knockdown. These data are in line with an inflammatory driver for iNOS production that is independent of the LFA-1/ICAM-1(ICAM-2/ICAM-3/ICAM-4/JAM-1) axes. Moreover, the reduction in NO production within the metastasis microenvironment suggests that the reduction in eNOS activity outweighed any increase in iNOS activity.

NO can have both anti- or pro-tumourigenic properties in cancer biology, depending on the type of tumour, its genetic background, the duration and level of exposure to NO, the site of production (tumour cells *vs.* host tissue) and which enzyme isoform prevails [21]. In some primary tumours, such as colon adenocarcinoma or lymphoma, tumour-derived NO promotes tumour progression [34, 35], primarily through an up-regulation of iNOS leading to a more proliferative and invasive tumour phenotype due to impairment of p53-induced apoptosis [35, 36]. In contrast, host-derived NO in ovarian cancer has been shown to inhibit tumour growth, depending on the levels of NO [37], and it has also been shown to exhibit protective effects on mesencephalic cells [38]. Here, our findings suggest that, in the context of brain metastasis, the high level of NO detected around the metastatic colonies in animals injected with parental MDA231Br-GFP cells is pro-tumorigenic.

It has been shown that low dose exposure to NO in certain types of cancer activates p53 and leads to tumour cell apoptosis; this effect likely results from induction of DNA damage by NO and its derivatives [39]. In accord with this, we found a marked increase in p53-DINP1 expression in mice injected with LFA-1 knockdown MDA231Br-GFP cells, together with significantly reduced tumour growth. MDA-MB-231 cells, are the progenitors of MDA231Br cells, and possess high levels of mutant p53 (mp53) which can contribute to the suppression of apoptosis in deprived conditions [40]. However, mp53 can also elicit cell cycle arrest and apoptosis in more efficient ways than wild-type p53 (wtp53) in other tumour cell types [41, 42]. In the current study, reduced levels of NO in animals injected with LFA-1 knockdown cells were associated with reduced metastasis growth and an increase in p53-DINP1. We were unable to differentiate between mp53 and wtp53, but the measured increase in p53 expression coupled with reduced tumour growth may suggest an mp53-driven and/or wtp53-driven pro-apoptotic gain-of-function. We also cannot exclude the possibility that increased nuclear p53 expression in tumour cells forced a negative feedback-loop on eNOS expression, thus reducing NO-induced DNA damage [39].

Taken together, our findings suggest the potential for targeting the LFA-1/COX-2/NOS axis therapeutically in brain metastasis. Anti-LFA-1 therapies have been taken into clinical trials for a number of conditions, including psoriasis [43], transplant rejection [44] and dry-eye syndrome [45]. However, no studies to date have pursued LFA-1 as a therapeutic target in either primary or secondary brain cancer. In contrast, a number of studies have demonstrated that tumour growth can be altered by inhibition of NOS, both *in vitro* and *in vivo*, although most of these studies sought to modulate iNOS rather than eNOS activity [46, 47]. Nevertheless, NO inhibitors have been shown to reduce tumour angiogenesis and blood flow in tumour-bearing mice [48]. Other studies have shown that the combination of COX-2 and NOS inhibitors reduces tumour growth in colon cancer [49], whilst patients on daily aspirin (non-selective COX inhibitor) suggest a reduced long-term risk for both primary and secondary cancers [50, 51]. Similarly, specific COX-2 inhibitors have been shown to induce apoptosis in lung carcinoma cells and reduce invasiveness in liver metastasis [52, 53]. Our data strongly support the concept that such strategies blocking NOS (e.g. L-NAME) and/or COX-2 (e.g. NS-398) may be highly beneficial in the treatment of brain metastasis.

In summary, we have demonstrated that disruption of tumour-host interactions via LFA-1 knockdown in tumour cells, dramatically reduces breast cancer metastasis within the brain. Moreover, this significant reduction in growth is associated with a decrease in COX-2 expression, primarily in astrocytes, and a downstream reduction in endothelial NO production. These findings suggest a potential scenario whereby under normal conditions, cross-talk between tumour cells and the brain parenchyma, by LFA-1 activated pathways, leads to increased COX-2 activity, VEGF expression and endothelial NO release, with a downstream reduction in tumour p53 levels (illustrated in Figure 7C), which warrants further investigation. LFA-1 was upregulated in human brain metastasis tissue from both breast and lung cancer patients supporting the clinical relevance of our findings. Together, these data provide strong evidence for pathways triggered by LFA-1 as a potential target for treating brain metastasis, and suggests the potential not only for anti-LFA-1 treatments, but also pre-existing anti-COX-2 or anti-NOS therapies.

## MATERIALS AND METHODS

### *In vivo* models of brain metastasis

All experiments were approved by the UK Home Office. Female BALB/c mice were anaesthetised with 2–3% isoflurane in 70% N_2_:30% O_2_, placed in a stereotactic frame and focally microinjected in the left striatum (+0.5 mm and 1.5 mm lateral from Bregma; depth 2.5 mm) with 5 × 10^3^ 4T1-GFP cells, a syngeneic metastatic mouse mammary carcinoma cell line, in 0.5 μl PBS using a 75 μm-tipped glass microcapillary (Clark Electromedical Instruments, UK). Animals were perfused at day 5, 10, 21 or 35 after tumour cell injection (*n* = 6 per time-point per group). To assess conservation of CAM expression across different tumour cell lines, and potentially extrapolate the data into the clinic, 5 × 10^3^ MDA231Br-GFP tumour cells, a metastatic human breast carcinoma cell line (kind gift from Dr P. Steeg, USA), were injected into female SCID mice as above (*n* = 5); animals were perfused at a single time-point, 14 days post-injection. Control BALB/c and SCID mice were injected with 0.5 μl saline, into the left striatum, as above (*n* = 3).

### Immunohistochemical and immunofluorescent analysis of mouse tissue

At the end of each experiment animals were transcardially perfusion-fixed under terminal anaesthesia with 0.9% heparinised saline followed by 200 ml of periodate lysine paraformaldehyde (PLP) containing only 0.025% glutaraldehyde (PLP_*light*_). Brains were post-fixed, cryoprotected, embedded and frozen in isopentane at −40°C. Expression of ICAM-1 and LFA-1 was assessed immunohistochemically on eight sections per animal (*n* = 6 per time point and group). Briefly, sections were washed in phosphate-buffered saline (PBS; Thermo Fisher Scientific, UK; pH 7.4) and quenched using 1% hydrogen peroxide (Sigma Aldrich, UK) in methanol. Sections were blocked and then incubated for 16 h with the appropriate primary antibody: anti-LFA-1 (1:250, Abcam-186873-, UK) and anti-ICAM-1 antibody (1:400, Abcam-124759-, UK). After rinsing in PBS, slides were incubated for 1h with the appropriate secondary antibody: biotinylated polyclonal to rabbit raised in goat (1:200, Vector Labs, CA, USA). After washing with PBS, staining was detected using a standard DAB/hydrogen reaction and sections were counterstained with cresyl violet. Slides were mounted and coverslipped using DPX mounting solution (Thermo Fisher Scientific, UK). A minimum of 8 sections per animal (*n* = 6 per time-point), evenly distributed through the striatum, were used to quantify the presence of each CAM; expression was quantified as the percentage of tumour area covered using an unbiased algorithm in Imagescope, which detects positive brown pixels.

To identify cell populations involved in the metastatic process, immunofluorescent co-localisation of LFA-1 and ICAM-1 with endothelial, glial or tumour cells was assessed using secondary antibodies labelled with different fluorophores (AMCA or Cy3). Sections were incubated for 16 h at 4°C with primary antibodies to LFA-1 or ICAM-1 (as above), and to one of the following cell markers: anti-CD34 (1:200, Abcam, UK); anti-GFAP (1:300, Dako, Denmark); anti-Iba1 (1:200, Abcam, UK). Slides were rinsed with PBS and incubated for 30 min with the appropriate secondary antibody in TNB (Tris-NaCl-Blocking buffer): biotinylated polyclonal antibody to rabbit (1:200, Vector Labs, CA, USA), biotinylated polyclonal to rat (1:200, Vector Labs, CA, USA) or biotinylated polyclonal antibody to goat (1:200, Vector Labs, CA, USA). Sections were then washed with PBS, incubated with streptavidin-HRP (PerkinElmer; 1:200) in TNB for 30 min, washed and incubated for 8 min in the dark with TSA-biotin (PerkinElmer; 1:100) in amplification buffer (PerkinElmer). Slides were washed and incubated with a streptavidin-A488 fluorophore (Invitrogen; 1:100) for 30 min. To detect the other fluorophore, the Texas Red secondary antibody (Vector Laboratories) was added at the same time as streptavidin-A488. Slides were counterstained using DAPI 1:1000 (Vector Laboratories) and cover-slipped using Vectashield mounting medium (Vector Laboratories).

To detect the cellular source of COX-2, immunofluorescent co-localisation of COX-2 with different brain cell populations was assessed within and around metastasis areas, for the MDA (*n* = 4) and KD#1 (*n* = 4) groups. Co-localisation of COX-2 presence with astrocytes (GFAP, 1:300, DAKO, UK), neurons (NeuN, 1:500, Millipore, USA), vessels (CD34, 1:200, Abcam, UK) and macrophages/microglia (F4/80, 1:100, Abcam, UK) was quantified using 5 intra- and peri-tumoural ROI per animal. The anti-COX-2 antibody (1:100, Abcam, UK) was tagged with Alexa488 (1:200, Vector, UK), and a Texas Red fluorophore (1:100, Vector, UK) was used for the rest of the antibodies.

All immunofluorescent images were acquired using an inverted confocal microscope (LSM-710; Carl Zeiss Microimaging) and analysed using ImageJ (NIH). Detection ranges were set to eliminate crosstalk between fluorophores: 409–485 nm for AMCA, 494–553 nm for GFP and 561-595 nm for Texas Red. For COX-2 analysis, an in-house plugin was designed to calculate the co-localisation coefficients m1 (red) and m2 (green), before quantifying co-localisation between each pair of fluorescent markers, as previously described [54]. The plugin was designed to determine the percentage of co-localisation between COX-2 staining (red) and each of the other cell markers (green), together with the percentage of area (within the image) of each marker that is above the user-defined threshold. All images were acquired at the same time and under the same conditions.

Nitrotyrosine (NT) was used as a marker of nitrated proteins and cell damage owing to the specific presence of nitric oxide (NO). At the same time, owing to its reported link with NO [21–23], levels of p53 expression were also quantified. To this end, 8 sections, spanning the region of metastasis, for each animal (*n* = 4 per group) were stained using an anti-NT antibody (1:300, Merck Millipore, Germany) and analysed in ImageScope using an algorithm that detects the number of brown pixels per section. NT area was normalised to tumour area for each brain metastasis assessed. A further 8 sections, adjacent to those used for NT analysis, were stained with an anti-p53 damage induced nuclear protein (DINP) antibody (1:300, Santa Cruz biotech, H-110, USA) and the presence of p53 quantified as a percentage of tumour area using ImageScope.

### Immunohistochemical analysis of human tissue

Human brain metastasis resection samples, from breast carcinoma (*n* = 2) and lung adenocarcinoma (*n* = 3) patients, were obtained from the leading edge of brain metastases, as described previously [55]. All samples came from an approved Biobank (Walton Research Tissue Bank; National Research Ethics Service #11/WNo03/2). Sections from each primary tumour (4 μm formalin fixed paraffin-embedded) were dewaxed in xylene and rehydrated in decreasing concentrations of ethanol in water and then stained for LFA-1 and ICAM-1 as above, using anti-LFA-1 (1:300, Abcam, UK) and anti-ICAM-1 (1:300, R&D systems, USA) antibodies.

### Modulation of LFA-1 expression

Since LFA-1 is constitutively expressed in both tumour cell lines used above, we chose to assess the role of LFA-1 in the human cell line (MDA231Br-GFP) to increase the translational relevance of our findings downstream. To determine the role of this protein during tumour growth shRNA was used to knockdown LFA-1 gene expression. Four different shRNA expression vectors were cloned in a pRFP-C-RS plasmid under the U6 promoter for mammalian cell expression specifically designed to bind ITGAL (CD11a) gene (Origene, USA). The shRNA plasmid was distinguished by puromycin resistance, and by red fluorophore (RFP) expression. Two different control plasmids were also produced; a purified and sequence-verified plasmid without shRNA cassette insert (shE) and a non-effective 29-mer scrambled shRNA cassette (shS).

Cells were grown to 80–90% confluence in DMEM medium containing 10% fetal bovine serum and 1% L-glutamine at 37°C, with 5% CO_2_. At this point, cells were digested with 0.25% trypsin solution containing 0.01% EDTA, centrifuged at 1200 rpm for 5 min and plated onto a 6 well with 5 × 10^5^ cells per well. The plates were incubated at 37°C with 5% CO_2_ overnight, and then the 6 groups of recombinant plasmid were transfected into the cells using Lipofectamine 2000 transfection reagent (Invitrogen, Carlsbad, CA) at a ratio of 1:1. After 6 h, RFP activity of the cells was detected by Leica fluorescent microscope (Olympus, Tokyo, Japan).

Following transfection of MDA231Br-GFP cells with shRNA against the LFA-1 gene, knockdown of protein expression was assessed by western blot. Total protein was extracted from five experimental cell lines; parental MDA231Br-GFP (MDA); shE; shS; and two clones with significant LFA-1 knockdown (KD#1 and KD#2). Cells were lysed in buffer containing 150 mM NaCl, 50 Mm Tris HCl pH 7.5, 5 mM EDTA pH 8.0, 1%Triton X-100, 0.1% SDS and protease inhibitors (cOmplete mini EDTA free, Roche). Protein concentrations for each sample were determined using the BCA protein assay kit (Fisher Scientific) according to the manufacturer's instructions and equal amounts were loaded on 10–12% TGX pre-cast gels (Mini –PROTEAN, Bio-Rad Laboratories, UK). Proteins were separated by SDS PAGE under reducing conditions and transferred onto PVDF (Immobilon-P, Fisher Scientific) or nitrocellulose (Whatman Protan, GE Healthcare) membranes. Membranes were blocked in 5% non-fat milk in PBS and incubated overnight at 4°C with anti-CD11a (1:1000, Abcam) or anti-alpha tubulin (1:2000, DM1 eBioscience) antibodies. Proteins were detected using appropriate HRP conjugated secondary antibodies (Sigma, UK) and a chemiluminescent substrate system (Super Signal West Pico, Thermo Scientific). The mean intensity of each band was quantified with respect to the parental MDA231Br-GFP cell line. Band densitometry was normalised to the intensity of tubulin bands and performed in triplicate.

To determine whether LFA-1 downregulation determined by Western blot reflected reduced expression of LFA-1 on the tumour cell surface, all experimental tumour cell lines were incubated with an anti-LFA-1 antibody (1:500, -MEM-83- Abcam, UK) for 30 minutes and then fixed and stained for LFA-1 detection (Supplementary Figure S7A–S7B). Further, stability of LFA-1 knockdown at later time points was assessed in MDA231Br-GFP cells after 7 passages (equivalent to day 20 after transfection) and retention of the plasmid (red fluorophore) was evident (Supplementary Figure S7C).

### MTT assay

To determine whether LFA-1 knock down in MDA231Br-GFP cells altered, the activity or proliferation of the different clones, we ran an MTT assay (Sigma-Aldrich, Germany). Four different clones used in this work (MDA, shE, KD#1 and KD#2) were seeded in a 96 well plate at 5.000 cells per well. Every experimental group was measured in a spectrophotometer (absorbance λ570) at days 1, 2, 3, 4, 5 and 10 after seeding. At day 5 all clones were at ~100% confluency. Therefore, cells were plated again on a different 96 well plate and measured 5 days later (day 10) to check that activity remained intact.

### *In vivo* assessment of the effect of LFA-1 knockdown on tumour growth

Five different sub-types of the MDA231Br-GFP cell line (MDA, shE, shS, KD#1 and KD#2) were injected intracerebrally in SCID mice, as described above. At day 14 and 21 after intracerebral tumour cell injection, animals were transcardially perfusion-fixed and the brains processed for histology as described above. To quantify tumour growth, 10 μm thick sections were cut across the entire striatum. Rostro-caudal growth of the tumours was determined from the distance between the most rostral and caudal sections containing tumour cells. All data were analysed blind to experimental conditions. To further assess tumour growth ImageScope software (Leica Microsystems, UK) was used to quantify tumour area for each animal (*n* = 6 per group) on at least 8 sections evenly distributed through the striatum. Tumours were circumscribed and compared to the total brain area analysed.

### Effect of LFA-1 knockdown on downstream signalling pathways

To assess the effect of LFA-1 knockdown on the expression of genes downstream of LFA-1 signalling, mRNA levels of molecules involved in either LFA-1 or ICAM-1 (as the predominant ligand to LFA-1) activation pathways were quantified in brain tissue 14 days after tumour cell injection. Five groups of animals (*n* = 5 per group) were injected into the left striatum, as above, with MDA, shS, KD#1, KD#2, or PBS. Seven different genes were assessed: CXCL12, CXCR4, TGF-β, VEGF, COX-2, eNOS and iNOS. Five of the target genes were analysed for both human (h) and mouse (m) origin to determine whether changes observed reflected production by tumour cells (human mammary carcinoma) or host tissue (SCID mouse brain). Thus, 12 genes were assayed in total. Since no tumour growth changes were found between the two control shRNA groups (shE/shS), shS was used as the control group for all further experiments. Since no changes were found in any of the human genes studied, we further assessed the sensitivity of our technique by measuring the expression of a gene, vimentin, known to be highly expressed in the parental MDAMB231 cell line [56]. Human vimentin levels were quantified following the same protocol as above.

Quantitation of mRNA expression was performed with 5 μl of cDNA, in a final volume of 20 μl containing 10 μl SYBR Green master mixture from PrimerDesign Precision (UK) and 300 mM sense and antisense primers (See Table 2). RT-PCR was performed in a Stratagene RT-qPCR machine (Agilent, UK) with MxQPCR software (Agilent, UK), using the following amplification protocol: enzyme activation 10 minutes at 95°C, denaturation 15s at 95°C and data collection 1 min at 60°C. During the data collection phase, fluorogenic data was collected through the 6-carboxyfluorescein (FAM) channel, which detects Syber green. A melt curve was also acquired at the end of the cycling. For mouse genes, qRT-PCR data were analysed in terms of fold expression in the four different MDA231Br cell lines used relative to averaged PBS injected control tissue. For the human genes, gene expression was compared with parental MDA groups. In both cases, the following equation was used:

$$\text{Fold expression}=\frac{{\left(\text{Efficiency GOI primer}\right)}^{\text{Control GOI Ct value}-\text{Experimental GOI Ct value}}}{{\left(\text{Efficiency HKG primer}\right)}^{\text{Control HKG Ct value}-\text{Experimental GOI Ct value}}}$$

**Table 2:** Sequences of RT-PCR oligonucleotide primers

GOI	Gene symbol	specie	Product length (b)	Melting T(°C)	Sense primer	Anti-sense primer
Chemokine receptor 4	Cxcr4	mus musculus	102	72	TACATCTGTGACC GCCTTTAC	AGGACGAGACCC ACCATTATAT
Chemokine ligand 12	CXCL12	homo sapiens	143	76.7	CTCCTCTTTCAACCT CAGTGATT	GAGAAGCAGAAGC AAGATTAAGC
Vascular endothelial growth factor A	Vegfa	mus musculus	116	74.2	ACTCTGCTAATGTTGG TGTCT	AAGCCTTTCAT CCCATTGTCTC
	VEGF	homo sapiens	93	73.4	CCAGGAAAGACTGAT ACAGAACG	GGTTTCTGGATTA AGGACTGTTC
Transforming growth factor, beta 1	Tgfb1	mus musculus	101	72.3	ATTAAAATCAAGTGTG GAGCAACAT	AGCGTATCAGTGG GGGTCAG
	TGFB1	homo sapiens	85	75.6	CATCAGAGCTCCGAGA AGCG	TCCACTTTTAACTTGA GCCTCAG
Nitric oxide synthase 3, endothelial cell	Nos3	mus musculus	105	73	CACTTCGTTCGGTTG ACCAA	AGGTAAACTGGA GAGAAGAAAG
	NOS3	homo sapiens	128	70	GAAGAACAAAGCTG AACATACTG	CTTCCCTGGAGTC TTGTGTAG
Nitric oxide synthase 2, inducible	Nos2	mus musculus	105	75	GTGTTCTTTGCTTC CATGCTAAT	GTCCCTGGCTAGTG CTTCAGAGA
	NOS2	homo sapiens	112	73	GAAGGCAGAGATCCA GGTGATGCA	CCTACAAGCCAAAG CAGTCTGTGA
Prostaglandin-endoperoxidase synthase 2	Ptgs2	mus musculus	123	73.7	TCATTTGAAGAACTT ACAGGAGAGA	GATAGCATCTGGAC GAGGTTTT
	PTGS2	homo sapiens	128	70	CAAATCAACACTGC CTCAAT	TCTGGATCTGGAA CACTGAATG

Where GOI = gene of interest, HKG = housekeeping gene (CANX and GADPH for mouse and human tissue, respectively) and Ct = number of cycles required for the fluorescent signal to cross threshold (background fluorescent levels).

The relative levels of individual mRNA in each sample transcript compared to a control housekeeping gene were calculated using the 2-^ΔΔ^Ct method, where ^ΔΔ^Ct = ^Δ^Ct sample – ^Δ^Ct reference. Here, ^Δ^Ct sample is the Ct value for any experimental sample normalised to the endogenous housekeeping gene and ^Δ^Ct reference is the Ct value for PBS samples also normalised to the endogenous housekeeping gene. Every experiment was run in duplicate and for the human genes, each gene was run twice on separate days.

### *In vitro* investigation of the VEGF/NO/p53 axis

To further probe the potential link between VEGF production within the tumour environment, endothelial NO release and p53 production by tumour cells, MDA231Br-GFP and KD#1 cells where co-cultured with either mouse (sEND1, kind gift from Dr. Robin Choudhury, UK) or human (hCMEC/D3; EMD Millipore SCC066) endothelial cells.

Endothelial cells were seeded at 1000 cells/well on a 24 transwell plate with 0,3 μm pores. 24 h later were incubated for 48 h with 20 ng/ml of recombinant VEGF or normal medium as control. After this time, a group of endothelial cells were assayed for NO production using a DAF-FM diacetate kit (ThermoFischer Scientific, US). Subsequently, the transwells with the cells were translocated onto a new 24 well plate in which either MDA or KD#1 tumour cells had been seeded at 5000 cell/well the day before. After 3 days of co-culture, tumour cells were harvested and p53 levels measured by western blot.

### Statistical analysis

Statistical analysis was performed using Prism (GraphPad Software Inc., USA). For the IHC quantitation, analysis of variance (ANOVA) was used to identify overall significant differences between the different time points or groups of animals, followed by Tukey post-hoc tests to identify specific differences between the groups. All data are expressed as mean ± S.E.M.

## SUPPLEMENTARY FIGURES


