# Supplementary material for: Disruption of tumour-host communication by downregulation of LFA-1 reduces COX-2 and e-NOS expression and inhibits brain metastasis growth
Source: Oncotarget. 2016 Jul 20;7(32):52375–91. doi: 10.18632/oncotarget.10737 (PMC5239559; doi:10.18632/oncotarget.10737)
Supplement: Supplementary file 1 [file oncotarget-07-52375-s001.pdf]

Disruption of tumour-host communication by downregulation of LFA-1 reduces COX-2 and e-NOS expression and inhibits brain metastasis growth

Supplementary Materials

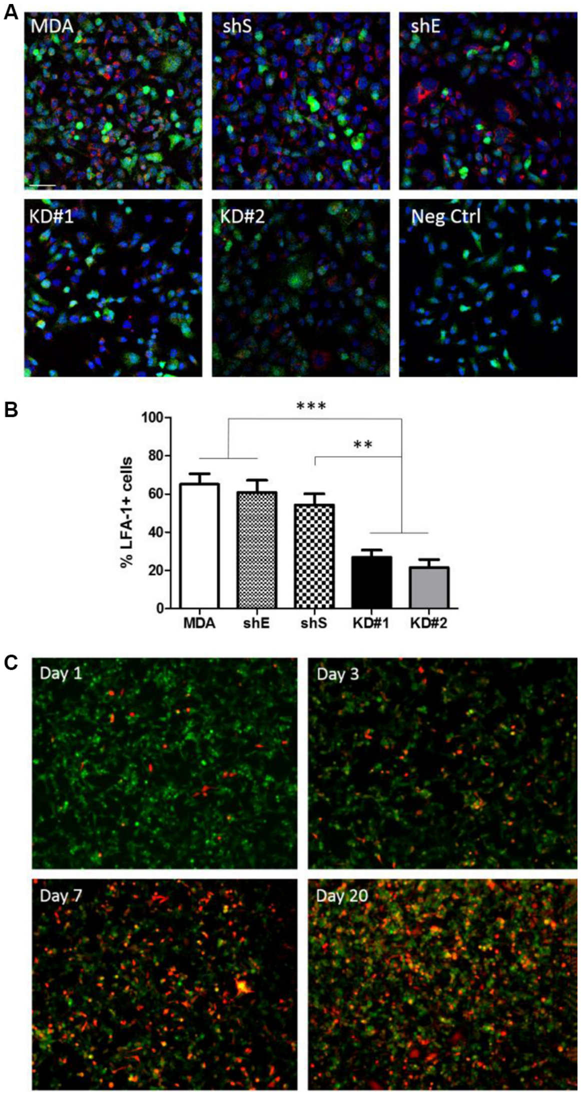

Supplementary Figure S1: (A) Assessment of cell surface expression of LFA-1. Cells were coated for 30 min with anti-LFA-1 antibody and then fixed for further staining. Immunofluorescent (red; Cy3 fluorophore) expression was evident on the cell surface of all the different clones used *in vivo* (MDA, shS and shE), but to a much lesser extent in the LFA-1 knock-down clones (KD#1 and KD#2). (**B**) Quantitation of LFA-1 positive cells per field of view; 4 different ROIs with > 300 cells were quantified for each group. (**C**) Assessment of shRNA transfection stability in MDA231Br-GFP cells (green). The shRNA plasmid against LFA-1 was coupled with red fluorescent protein (RFP, red) and puromycin resistance. Presence of the shRNA plasmid was determined at days 1, 3, 7 and 20 after the onset of transfection. The cells showed stable presence of the shRNA plasmid (red and green co-localisation) from day 7 to day 20 post-transfection. For the *in vivo* studies, transfected cells were injected intracerebrally at day 7 after transfection and tumour growth assessed 14 days later (≈ day 20 post-transfection). These data suggest, therefore, that the cells should maintain stable plasmid transfection throughout the *in vivo* time course studied.

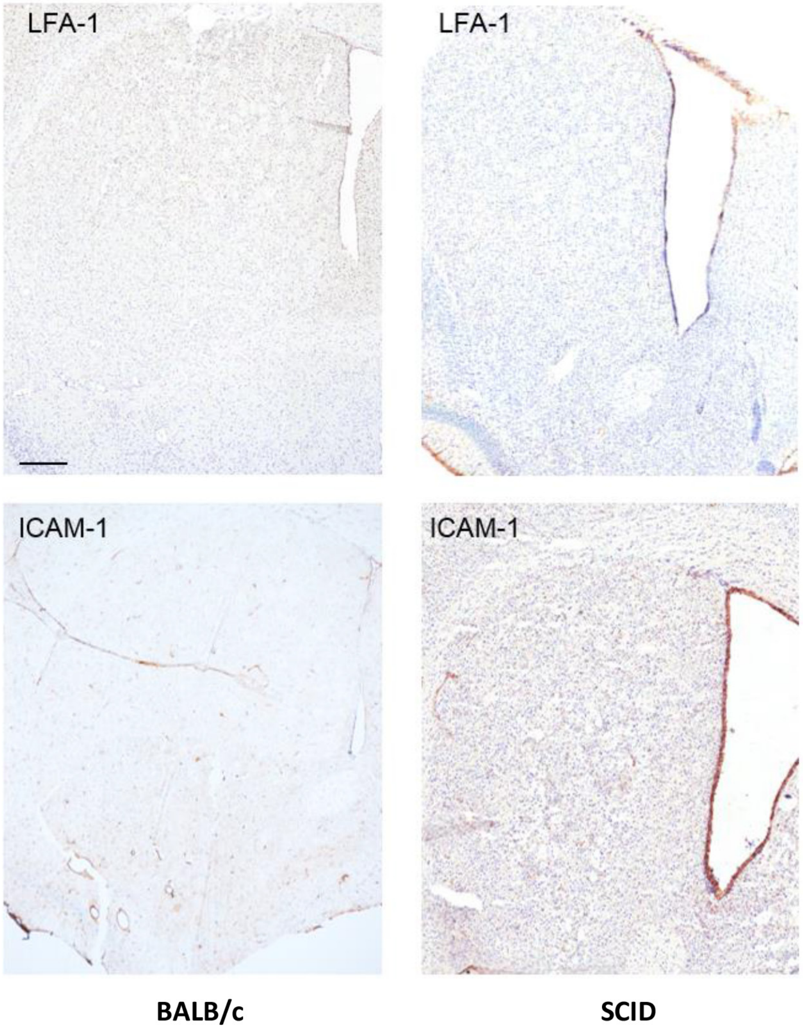

Supplementary Figure S2: Photomicrographs from the left striatum of BALB/c (left panels) and SCID (right panels) mice 10 and 14 days post-injection with PBS, respectively. Sections were stained against LFA-1 (top) and ICAM-1 (bottom). Basal levels of expression of both CAMs were significantly lower than the experimental groups and largely undetectable. Scale bar = 300 μm.

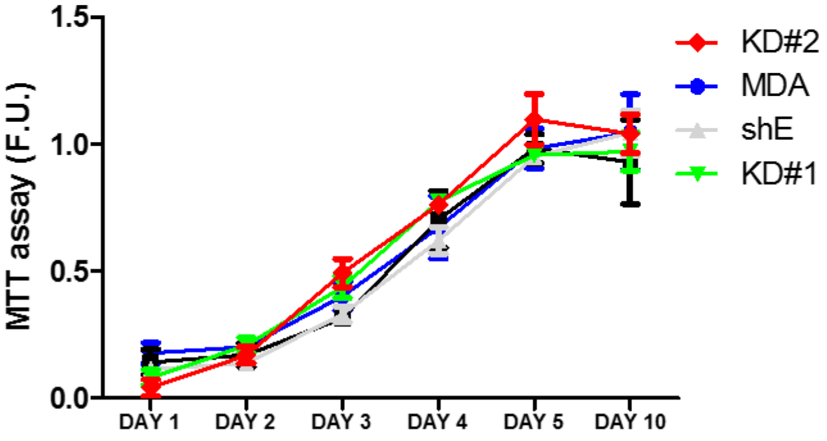

Supplementary Figure S3: MTT assay study of four different MDA231BR-GFP clones used in the *in vivo* study. No differences in cell activity were found between the parental cells and the LFA-1 knock-down clones.

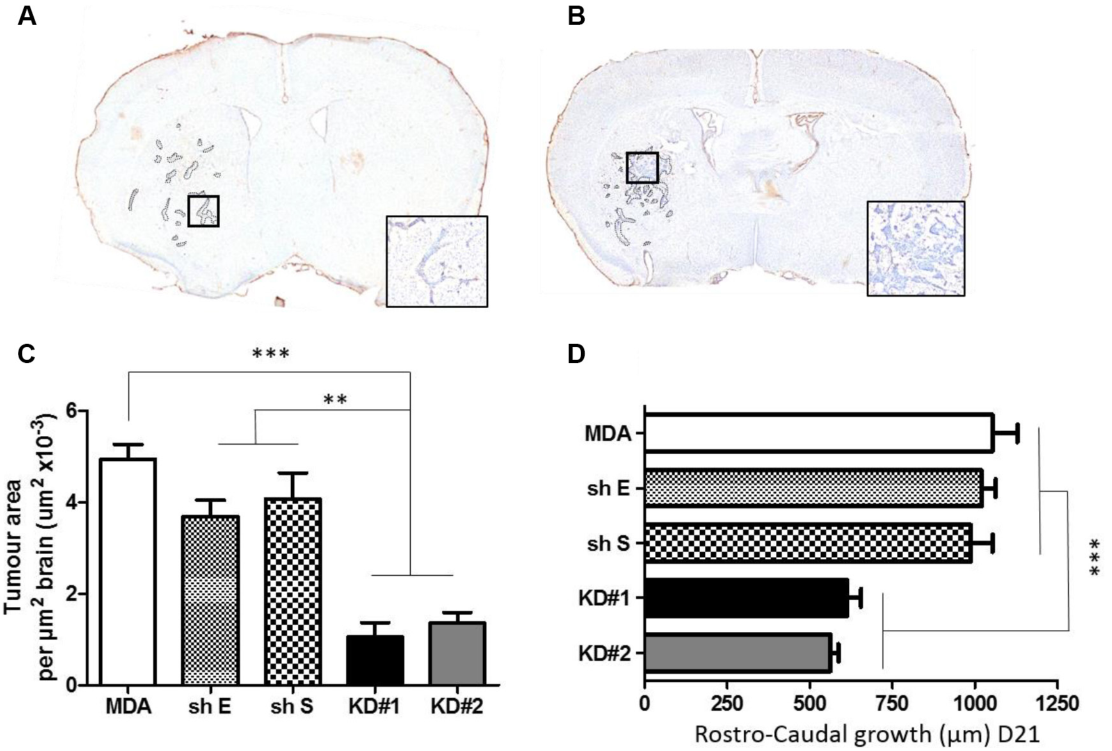

Supplementary Figure S4: (**A**–**B**) Photomicrographs of SCID mice 21 days after injection of KD#1 (a) and MDA (b) tumour cells, respectively. (**C**) Following the previous study of tumour growth at day 14 after tumour induction (Figure 3), quantitation of tumour growth at a later time point (day 21) was performed in animals injected intrastriatally with parental MDA231Br-GFP cells (MDA), control knock down cells (empty cassette, shE; and scramble cassette, shS) or LFA-1 knock down cells (KD#1 and KD#2) ($n = 4$ per group). Statistical significance was determined by one-way ANOVA, with Tukey's post-hoc tests. $^{**}p < 0.01$; $^{***}p < 0.005$ (**D**) Quantitation of rostro-caudal tumour growth throughout the striatum in the same animals as for (C). Statistical significance determined by one-way ANOVA, with Tukey's post-hoc tests. $^{***}p < 0.005$.

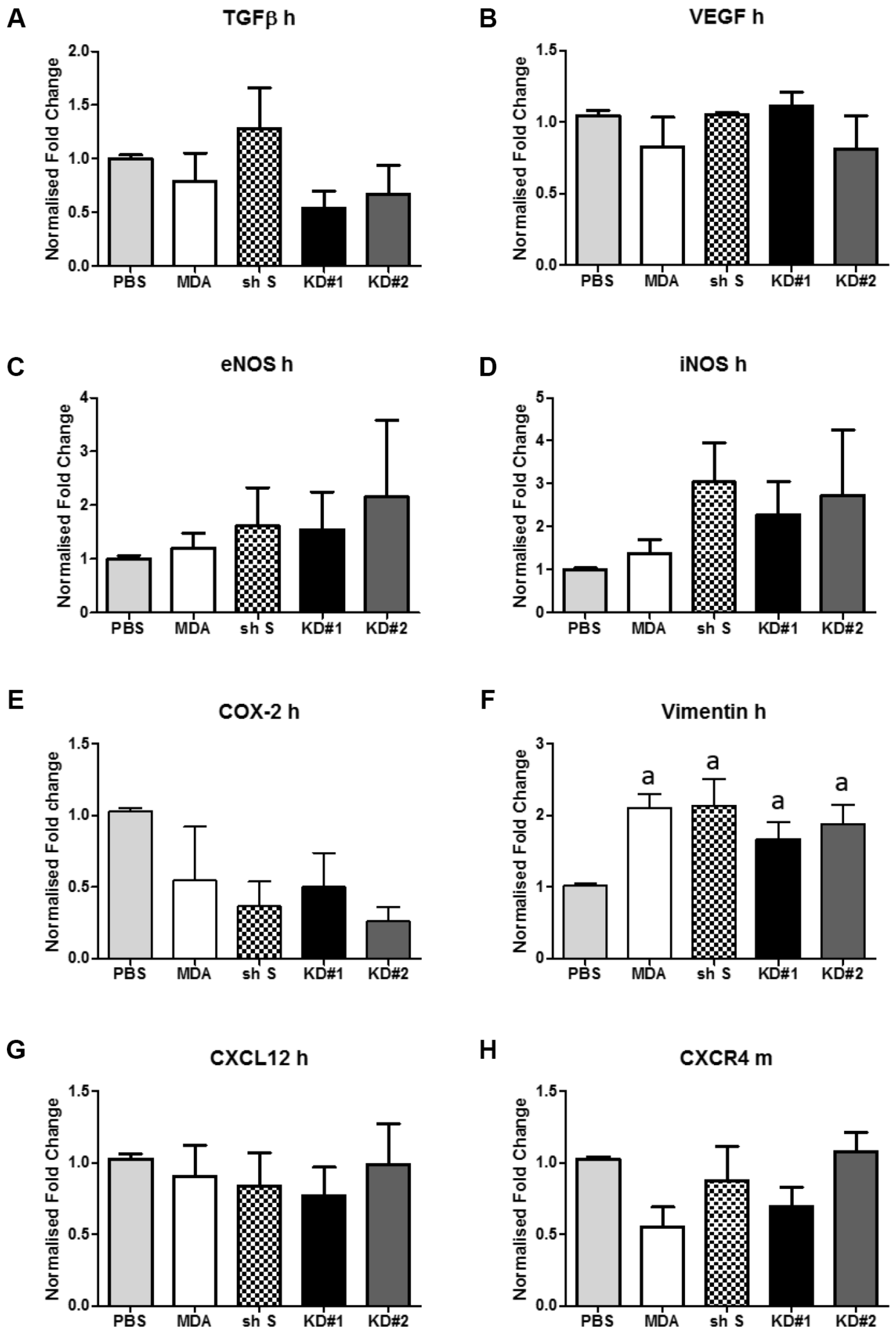

Supplementary Figure S5: Quantitative reverse transcription PCR analysis of (A–G) human and mouse (H) gene expression in the striatum of mice injected with either PBS or one of the MDA231Br-GFP clones ($n = 5$ per group). Statistical significance determined by one-way ANOVA, with Tukey post-hoc tests. Multiple Comparison Test: a= significant compared to the PBS group. $p < 0.05$

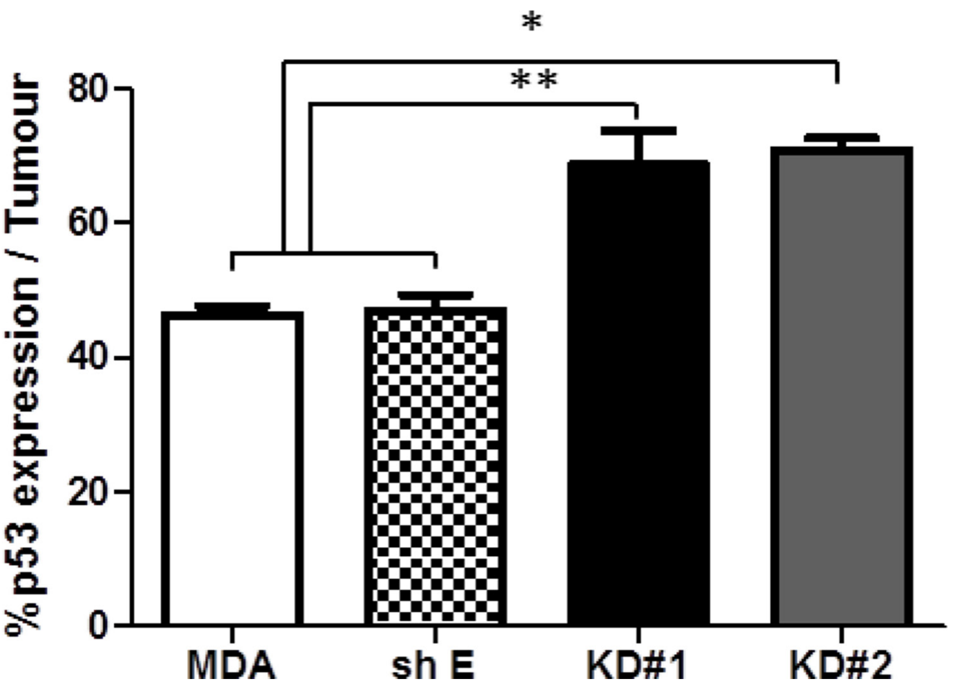

Supplementary Figure S6: Quantitation of p53 expression levels within tumour colonies 21 days after intracerebral injection of MDA231Br-GFP cells (MDA), control knock-down cells (shE) or LFA-1 knockdown cells (KD#1 and KD#2). Statistical significance was determined by one way ANOVA followed by Tukey's post-hoc tests; *$p < 0.05$ and **$p < 0.01$.

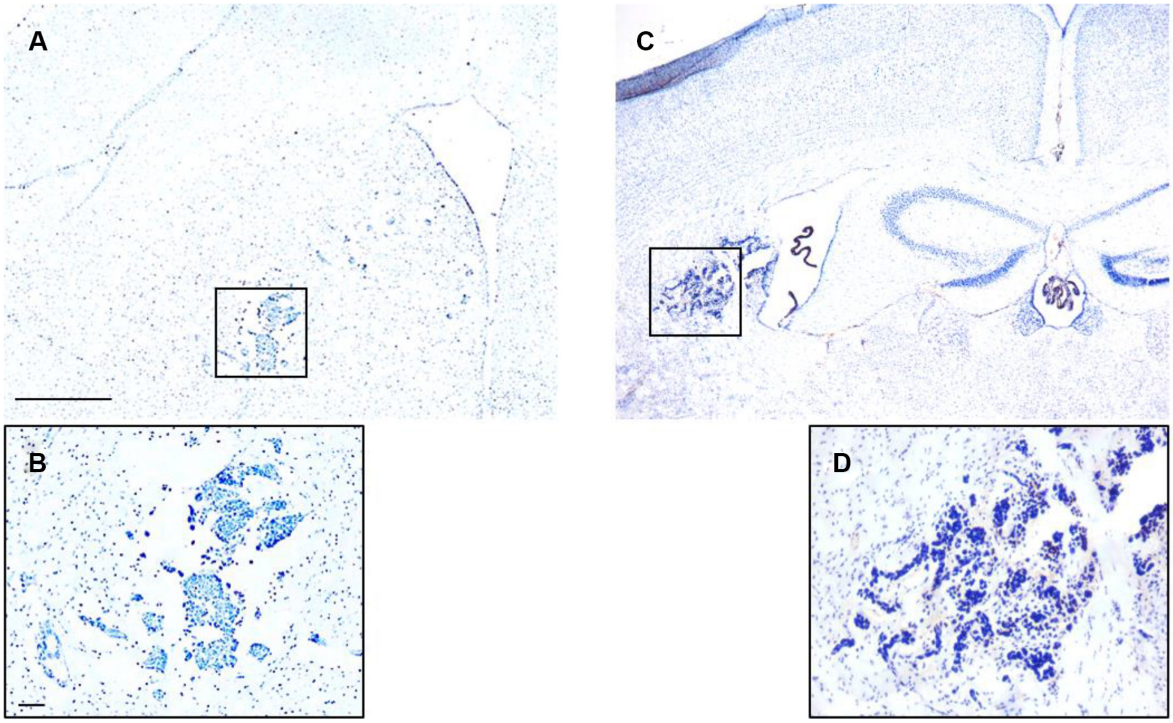

Supplementary Figure S7: MDA231Br-GFP cells injected into the left striatum of the brain (A), or left ventricle of the heart (C) in SCID mice. Insets show a similar pattern of growth in the tumour colonies induced either by direct intracerebral injection (**B**) or via haematogenous dissemination to the brain (**D**). Scale bar 500 µm and 50 µm (inset)
